# Transcriptome and metabolome analyses reveal that *Bacillus subtilis* BS-Z15 lipopeptides mycosubtilin homologue mediates plant defense responses

**DOI:** 10.3389/fpls.2022.1088220

**Published:** 2023-02-06

**Authors:** Qilin Yang, Hui Zhang, Jia You, Jun Yang, Qi Zhang, Jinjin Zhao, Reyihanguli Aimaier, Jingbo Zhang, Shengcheng Han, Heping Zhao, Huixin Zhao

**Affiliations:** ^1^ Xinjiang Key Laboratory of Special Species Conservation and Regulatory Biology, College of Life Science, Xinjiang Normal University, Urumqi, China; ^2^ Beijing Key Laboratory of Gene Resource and Molecular Development, College of Life Sciences, Beijing Normal University, Beijing, China

**Keywords:** plant-microbial interactions, systemic resistance, B. subtilis BS-Z15, mycosubtilin homologue, RNA-Seq, GC-MS

## Abstract

Microbial-plant interactions protect plants from external stimuli, releasing various elicitor that activate the plants defense response and regulate its growth. *Bacillus subtilis* BS-Z15 was screened from cotton inter-rhizosphere soil, antagonized various plant pathogens, and protected cotton against *Verticillium dahliae*. This study showed that the BS-Z15 lipopeptide mycosubtilin homologue could act as an elicitor to induce systemic resistance (ISR) in plants. Mycosubtilin homologue induced ROS burst and deposition, callose deposition, MAPK cascade phosphorylation, and up-regulated *PR1* and *PDF1.2* gene expression in *Arabidopsis* seedlings, moreover enhanced resistance of *Arabidopsis* to *Pseudomonas syringae* pv. *Tomato* DC3000 (*Pst* DC3000) and *V. dahliae.* Transcriptome analysis was then used to evaluate the impact of mycosubtilin homologue on plant gene expression control. Mycosubtilin homologues activated *Arabidopsis* ISR on genes in metabolic pathways such as *Arabidopsis* plant-pathogen interactions, phenylpropanoid biosynthesis, MAPK signaling pathway, and phytohormone signaling. These analyses revealed that mycosubtilin homologues mediate the regulation of plant systemic resistance and growth and development by affecting related metabolites in glycolysis and gluconeogenesis, pentose phosphate pathway, tricarboxylic acid cycle, and amino acid metabolism in *Arabidopsis*. These findings confirmed that a mycosubtilin homologue could trigger the initiation of the *Arabidopsis* ISR by interacting with a variety of PTI components and transcriptional metabolic signaling pathways.

## Introduction

Plants become surrounded by secondary metabolites released by microorganisms during growth and development (Kai et al., 2007). Some secondary metabolites may affect growth through different mechanisms, such as biochemical signals that elicit local defense responses or systemic resistance (Chung et al., 2016). Bacteria and plants, can interact through isoprene, terpenoids, alkanes, olefins, alcohols, esters, carbonyl groups and acidic lipopeptides (Kesselmeier and Staudt, 1999). Moreover, secondary metabolites of beneficial microorganisms can promote plant growth and induce systemic resistance (ISR) (Ryu et al., 2003; Choudhary et al., 2007; Zheng and Dong, 2013). The mechanisms involved in microbial-plant interactions are diverse and include competition for nutrients, ecological niches, antagonism, production of secondary metabolites to aid host plant growth, and immune regulation (Ongena et al., 2007; Romero et al., 2007). The production of Bacillus isolates overexpressing bacteriophage manipulator synthesis was studied, which affects 2,3-butanediol levels. The overexpressing strain was found to be more persistent than the wild type in colonized pepper roots, preventing the colonization of *Mycobacterium avium* and *Ralstonia solanacearum*. *Penicillium avium* infection following 2,3-butanediol application to the roots enhanced the expression of pathogenesis-related genes (PR) (Yi et al., 2016). Volatiles triggered the secretion of root secretions that regulated the adaptation of soil fungi and bacteria, thus facilitating the induction of plant defenses (Kong et al., 2021).

Members of the genus Bacillus are usually capable of producing a large number of secondary metabolites, with the potential to produce more than 20 structurally distinct antimicrobial compounds (Emmert and Handelsman, 1999; Stein, 2005). These antimicrobial compounds are mainly cyclic lipopeptides (LPs) such as iturin, surfactin, and fengycin. The different species of LPs can bring advantages to specific ecological niches (Ongena and Jacques, 2008; Dunlap et al., 2019). *Bacillus* LPs-like substances are involved in biocontrol activities against various pathogenic bacteria and plant species. Production of iturin A by *B. subtilis* RB14 alleviates the disease caused by *Rhizoctonia solani* (Asaka and Shoda, 1996; Zohora et al., 2016). The mycosubtilin produced by *B. subtilis* ATCC6633 inhibits the growth of *Fusarium graminearum* and *Fusarium verticillioides* and suppresses the expression of their toxin genes (Yu et al., 2021). LPs can also be used as inducers of plant resistance, inducing plants to develop resistance to pathogenic bacteria. Purified compounds also displayed equal protection to activity derived directly from production strains in cotton and beans, where the role of surfactin and fengycin in the induction of resistance in plants was revealed (Ongena et al., 2007).

Previous research has identified mycosubtilin homologues in secondary *B. subtilis* metabolites that can improve cotton’s systemic resistance. In the present work by studying the role of mycosubtilin homologue on *Arabidopsis thaliana* at the physiological, biochemical, cellular, molecular and transcriptional metabolome levels, we sought to understand the molecular mechanisms by which mycosubtilin homologue regulates plant defense responses. The bioinformatic network of mycosubtilin homologue-induced systemic resistance in plants was constructed by transcriptome (RNA-Seq) and metabolome (GC-MS), the main differential genes, differential metabolic pathways, and differential metabolites of the substance-induced systemic resistance in *Arabidopsis* were screened. This study establishes a foundation for *Bacillus subtilis* and its secondary metabolites’ coordinated regulation of plant systemic resistance, growth, and development. Additionally, it directs the usage of *B. subtilis* and its mycosubtilin homologue secondary metabolites for biological control.

## Materials and methods

### Strain recovery and preparation of mycosubtilin homologue

The fermentation broth of BS-Z15 was briefly collected by centrifugation to remove bacterial residues and adjusted to pH 2.0 with hydrochloric acid at 4°C overnight according to a previously described method (Lin et al., 2022). The acid residue was then collected by centrifugation at 4,000g and dissolved in pre-cooled (-20°C) acetone at 80% (v/v) for 4 hours. The acetone-extracted supernatant was air dried and dissolved in water. The upper butanol phase was obtained, mixed with an equal amount of n-butanol, and then collected and evaporated. The mixed compounds were dissolved in DMSO and cleaned up using a semi-preparative HPLC system (C18, 5 μm, 250×10 mm, Hypersil GOLD^™^, CA). The chromatography was monitored at 215 nm using gradient elution with 40%-50% acetonitrile (0.05% TFA, v/v) at a flow rate of 2 mL min^-1^.

### Culture of plant and bacterial material

The wild-type Columbia-0 (Col-0) *Arabidopsis* plant lines used in this study were cultivated in a controlled growth environment at a temperature of 23 ± 1°C and 70% relative humidity with a 16 h light and 8 h dark photoperiod. For the MAPK activity assay, transcriptome sequencing, and metabolome, Murashige and Skoog media were utilized to culture 10-day-old *Arabidopsis* Col-0 seedlings. The remaining trials in this study were conducted consistently with soil-grown plants that were 4 weeks old.

*Pst* DC3000 grown in liquid King’s B medium (BD Difco) containing, per litre, 50 mg of rifampicin and 50 mg of kanamycin at 37°C overnight. Bacterial cells were pelleted by centrifugation and resuspended in 10 mM MgCl_2_. *V. dahliae* was grown in a Czapek-Dox medium (BD Difco) at 28°C for 48 h. The fungal spores were isolated through a filter membrane and resuspended in 10 mM MgCl_2_.

### Detection of ROS. burst and callose callosum deposition

ROS production was measured as reported previously by (Smith et al., 2014). Briefly, leaf discs (0.2 cm^2^) were cut out from *Arabidopsis* with a punch and incubated overnight in water in 96-well plates. The following day, 200 mM luminol, 20 mg/mL horseradish peroxidase, 100 nM flg22 and mycosubtilin homologue (1 μg/mL and 10 μg/mL) were added to each well.

*Arabidopsis* Col-0 leaves that were 4 weeks old were syringe-infiltrated with water and 10 μg/mL of mycosubtilin homologues (the control). The leaves were taken 0, 6, and 12 hours after the inoculation to find ROS. Diaminobenzidine (DAB, pH=3.0) was used to stain *Arabidopsis* leaves for 8 hours while they were left in the dark and at room temperature. After 30 min of incubation in a solution of ethanol, acetic acid, and glycerol (3:1:1, vol/vol/vol), leaves were gently rinsed with water before being examined under a light microscope. DAB staining revealed hydrogen peroxide as a reddish-brown precipitate.

Leaves of 4 week old *Arabidopsis* Col-0 plants were syringe-infiltrated with 10 μg/mL of mycosubtilin homologue and water (the control). To determine callose deposition, leaves were immersed in 5 ml of destaining solution (phenol/glycerol/lactic acid/water/ethanol, 1:1:1:1:8, vol/vol/vol/vol/vol) in a bottle. They were infiltrated by applying a vacuum for 5 to 10 min. The bottle with samples was incubated in a 90°C water bath for 30 min to clear chlorophyll. The resulting chlorophyll-free leaves were then gently rinsed with water before being stained for 2 to 4 hours in the dark with 0.01% (wt/vol) aniline blue staining solution containing 150 mM K_2_HPO_4_ (pH=9.5) and preserved with 50% glycerine. After staining, leaves were put on microscope slides and gently washed with water before being examined with an epifluorescence microscope fitted with a UV excitation filter.

### MAPK activity assay in *Arabidopsis* seedlings

Seedlings grown on Murashige and Skoog medium for two weeks were sprayed with μ10 g/mL of mycosubtilin homologue. Samples were collected at 0, 5, 10, and 30 min and were analyzed by Western blotting using monoclonal rabbit phospho-p44/42 MAPK (Erk1/2) XP antibodies (Cell Signaling Technology, Danvers, MA, USA) (1:2,000 dilution). Detected with a monoclonal mouse anti-α-tubulin (Sigma-Aldrich) (1:4,000 dilution).

### RNA extraction and RT-qPCR analysis

Leaves of four week old *Arabidopsis* Col-0 plants were syringe-infiltrated with 10 μg/mL of mycosubtilin homologue and water (the control). Leaf tissues were sampled at 2 h, 4 h, 6 h, 12 h, 24 h, 48 h, 72 h, and 144 h, from which RNA was extracted using TRIzol reagent (Invitrogen, San Diego, CA, USA). Total RNA was treated with DNase I (Invitrogen). cDNA was synthesized from 1 µg of RNA using the SuperScript III first-strand synthesis system (Invitrogen). qRT-PCR was performed using SYBR Green JumpStart Taq ReadyMix (Sigma, St. Louis). *Arabidopsis* Actin was used as a control to normalize gene expression levels. **Supplementary Table 1** lists the primers used for real-time PCR.

### Fungi and bacterial infestation of *Arabidopsis*

A 50 μg/mL of mycosubtilin homologue and water were syringe-infiltrated into 4-week-old *Arabidopsis* seedlings (the control) leaves (Niu et al., 2016; Nie et al., 2017). The leaves were challenge-inoculated by syringe infiltration with a suspension of *V. dahliaee* and *Pst DC3000* (courtesy of the School of Life Sciences, Beijing Normal University) at 10^6^ CFU/mL concentration one day after pretreatment. At least 12 leaf discs were collected for each growth assay, and pathogenic infection was observed two days later.

### Transcriptome sequencing (RNA-Seq) analysis process

Total RNA was extracted from the tissue samples, and the concentration and purity of the extracted RNA were measured using Nanodrop 2000, RNA integrity was measured by agarose gel electrophoresis, and RIN values were determined by Agilent 2100. Using magnetic beads with Oligo (dT) for A-T base pairing with ployA, mRNA can be isolated from total RNA and used to analyze transcriptome information. Using mRNA as a template, six-base random primers (random hexamers) are added in the presence of reverse transcriptase to synthesize one-stranded cDNA, followed by two-stranded synthesis. All mRNAs were sequenced using the Illumina Novaseq 6000 sequencing technology for eukaryotic mRNA sequencing. The Illumina TruseqTM R.N.A. sample prep Kit was utilized for library preparation in sequencing assays. Salmon was used for Unigene expression level analysis by calculating transcripts per million (TPM). DESeq2 software was used to analyze raw counts based on the negative binomial distribution, and genes/transcripts comparing expression differences between groups were obtained using certain standardized processing and screening conditions with default parameters: p adjust < 0.05, log_2_ fold change ≥ 1 and statistical significance (p < 0.05) by R package edgeR. Using DIAMOND and a threshold E value of < 0.00001 Pfam, unigenes were aligned against the non-redundant (Nr) protein databases Gene ontology (GO), SwissProt, Kyoto Encyclopedia of Genes and Genomes (KEGG), and eggnog. The previously published article (Ren et al., 2022) describes the analysis tools and procedures used in the paper.

### Metabonomics (GC-MS) analysis process

50 mg of sample was weighed into a 2 ml centrifuge tube, and 0.5 ml of methanol-water solution (CH3OH: H2O v: v=4:1, containing 0.02 mg/mL of standard internal L-2-chloro-phenylalanine) was added. Add steel balls and place them in a grinder at -20°C (50 Hz, 3 min). Add 200 μL of chloroform, grind in a grinder (50 Hz, 3 min) and extract with ultrasound for 30 min. Centrifuge at low temperature for 15 min (13,000 rcf, 4°C), remove the supernatant into a glass derivative vial and blow dry under nitrogen. 80 μL of pyridinium methoxide hydrochloride solution (15 mg/mL) was added to a glass derivative vial, vortex shaken for 2 min, and then oxidized for 90 min at 37°C in a shaking incubator. Followingremoval, 80 μL of BSTFA derivatization reagent (containing 1% TMCS) was added, the vortex was agitated for 2 min, and the reaction was performed at 70°C for 60 min. Samples were removed and left at room temperature for 30 min for GC-MS metabolomics analysis.

Gas chromatograph-mass spectrometer Agilent 8890B-5977B was the analytical device utilized in this experiment (Agilent, USA). The sample was divided on a DB-5MS capillary column (Agilent 122-5532G, 40 m x 0.25 mm x 0.25 m) before being subjected to mass spectrometric analysis. The sample inlet temperature was 260°C, the carrier gas was high purity helium, the carrier gas flow rate was 1 mL/min, the spacer purge flow rate was 3 mL/min, and the solvent delay was 5 min. The ramp-up procedure was 60°C for 0.5 min, followed by a ramp-up to 310°C at 8°C/min for 6 min. Electron bombardment ion source (EI), transmission line temperature 310°C, ion source temperature 230°C, quadrupole temperature 150°C, electron energy 70 eV. Scan mode is full scan mode (SCAN), mass scan range: m/z 50-500, scan frequency 3.2 scan/s. The downstream files were pre-processed with MassHunter workstation Quantitative Analysis (v10.0.707.0) software for peak extraction and alignment. The group datasets were normalized before the analysis was performed. The probabilistic quotient normalization algorithm was used to accomplish data normalization on all samples. Then, batch correction of QC-robust splines was carried out utilizing QC samples. The final metabolite identification findings and data matrix were merged using a *t-test* and VIP (OPLS-DA) to screen the differential metabolites (Ren et al., 2022).

### Statistical analysis

Statistical analyses were performed using the GraphPad Prism 8 software. Unpaired Student’s *t-test* determined the statistical significance. ns indicates no significant difference*, ** and *** specifies statistical significance at p< 0.05, p < 0.01 and p < 0.001, respectively.

## Results

### Mycosubtilin homologue enhance plant resistance to pathogens by regulating plant growth

**Figure 1:**
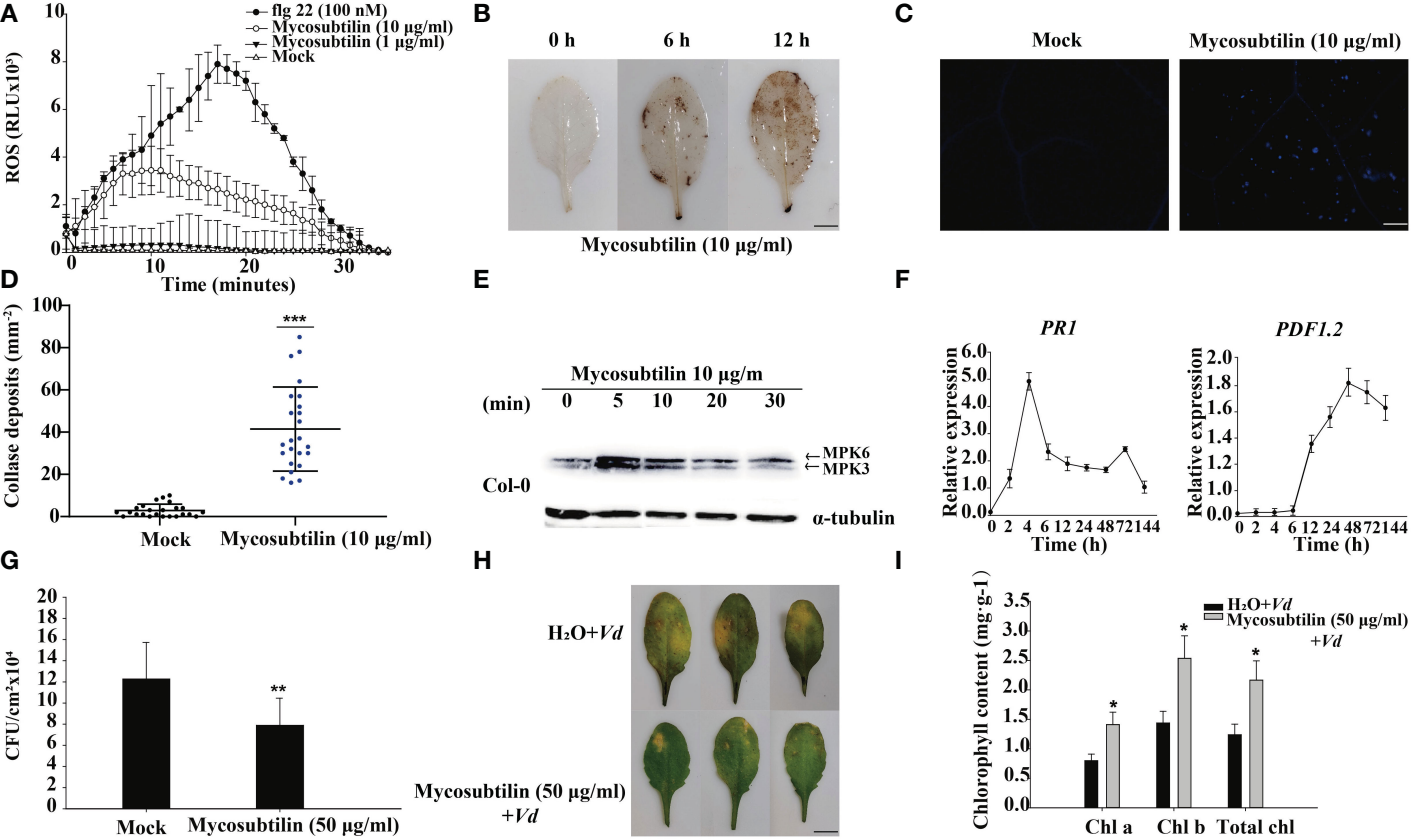
The early immune response of plants to mycosubtilin homologues. **(A, B)** Effects on the burst of reactive oxygen species in *Arabidopsis* leaves, means ± SD, n = 24, repeat = 3, scale bars 0.5 cm. **(C, D)** Effect on callose deposition in *Arabidopsis* leaves, n = 24, repeat = 3, scale bars 200 μm. **(E)** Mycosubtilin homologues activate MAPK signaling pathway in *Arabidopsis*; α-tubulin was used as an equal loading control. **(F)** Inducible expression of *Arabidopsis* resistance-associated genes *PR1* and *PDF1.2*, Means ± SD, n = 12, repeat = 3. **(G, H, I)** Mycosubtilin homologues protects to *Arabidopsis* against pathogenic bacteria, means ± SD, n = 24, repeat = 3, Scale bars 0.5 cm. *, ** and *** indicate statistical significance at p < 0.05, p < 0.01 and p < 0.001, respectively (Student’s t-test).

The results of a 10 day mycosubtilin homologue treatment of *Arabidopsis* seedlings revealed that at certain concentrations, mycosubtilin homologue inhibited main root growth while promoting lateral root development. The effect of mycosubtilin homologue on *Arabidopsis* growth and development was concentration-dependent, with higher concentrations resulting in more pronounced inhibition of *Arabidopsis* root growth, still this inhibition of growth was not lethal (**Supplementary Figure 1**). However, mycosubtilin homologue exhibits inhibitory but not lethal effects on *Arabidopsis* at very low concentrations. Therefore, to study this phenomenon, we examined the early immune response of plants. After treatment of *Arabidopsis* leaves with 1 μg/mL and 10 μg/mL mycosubtilin homologue, the luminous response showed that *Arabidopsis* produced ROS rapidly at 2 min and reached the highest value of around 10-15 min. This response finally lasted for about 30 min. Furthermore, the burst of *Arabidopsis* ROS was dependent on mycosubtilin homologue concentration (**Figure 1A**). *Arabidopsis* leaves 12 h after injection of 10 μg/mL mycosubtilin homologue, the red-brown precipitate was found in leaves by tissue staining, indicating that mycosubtilin homologue induced a large accumulation of ROS in *Arabidopsis* leaves (**Figure 1B**). In the aniline blue staining, a large amount of callose deposition around the cell wall of Arabidopsis leaves was observed after mycosubtilin homologue treatment, using fluorescence microscope (**Figures 1C, D**). In the treatment with mycosubtilin homologues, MPK3/6 phosphorylated protein activation was detected by pERK antibody and phosphorylated MPK3 and MPK6 were detected at 5 min and persisted until 30 min (**Figure 1E**). Using 10 μg/mL treatments of the mycosubtilin homologue, the expression of the *PR1* and *PDF1.2* genes in the four-week-old Arabidopsis SA (Salicylic acid), JA/ET (jasmonic acid/Ethylene) signaling pathway was investigated. Following treatment with a mycosubtilin homologue, both genes displayed increased expression. The expression of the *PR1* gene started to increase at 2 h after treatment, reached the highest level at 4 h, and continued to 144 h. The expression of the *PDF1.2* gene began to increase at 12 h after treatment, reached the highest level at 48 h, and continued to 144 h (**Figure 1F**). *Arabidopsis* leaves were treated with 10 μg/mL of mycosubtilin homologue injection for 12 h and then inoculated with *Pst* DC3000 and *V. dahliae*. The findings demonstrated that after being treated with mycosubtilin homologues, *Arabidopsis* exhibited moderate resistance to *Pst* DC3000, and the number of *Pst* DC3000 colonies in the treated *Arabidopsis* leaves was dramatically decreased (**Figure 1G**). *Arabidopsis* showed some resistance to *V. dahliae* after mycosubtilin homologue treatment, and the chlorosis and yellowing of treated *Arabidopsis* leaves were significantly reduced compared with untreated leaves. The damage to leaves was reduced after mycosubtilin homologue treatment, and chlorophyll content was significantly higher than in untreated leaves (**Figures 1H, I**).

### Transcriptome analysis of mycosubtilin homologue treated *Arabidopsis*

**Figure 2:**
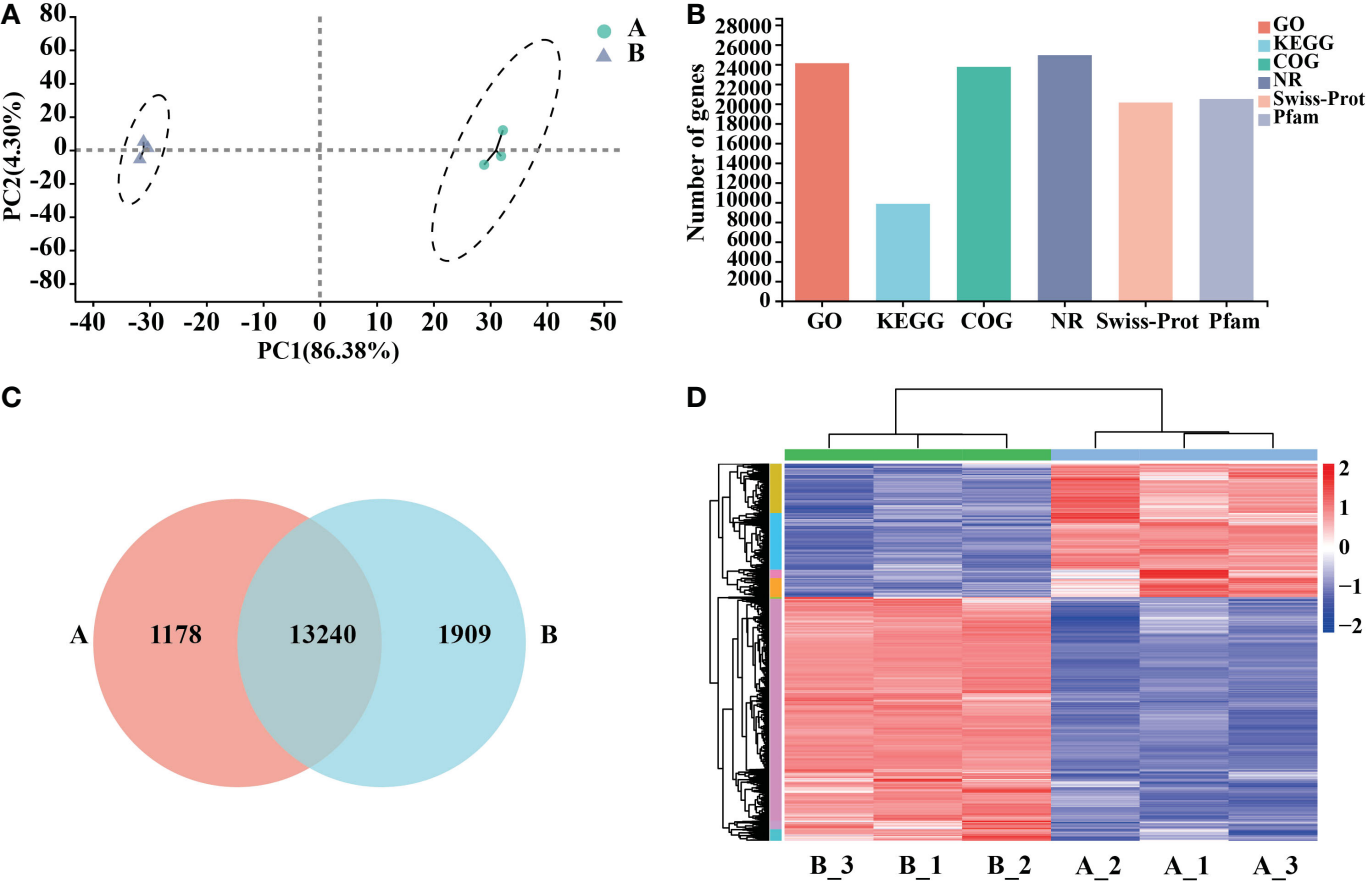
Functional annotation and comparative analysis of transcriptome data. **(A)** Inter-sample P.C.A. mapping. **(B)** Transcriptome database gene annotation. **(C)** Veen map of the differential gene. **(D)** Heat map for differential gene clustering.

Six samples of *Arabidopsis* total RNA were extracted at two treatment time points of 0 h and 12 h, high-quality RNA (**Supplementary Table 2**). The transcriptome was sequenced, and a total of 25,917 genes were detected, including 25,689 known genes and 228 novel genes; after filtering Raw reads, about 44 million clean reads were obtained from control (A) and processing (B) groups (50 μg/mL mycosubtilin homologue). All samples had Q20 above 98.4% and Q30 above 95%, with good GC content and base quality distribution (**Supplementary Table 3**). Downscaling was used to assess the data, and the intra-group correlations between the samples were strong, whereas the correlations between (A) and (B) were quite different (**Figure 2A**). The transcriptome data fromthe GO database(containing 24102 genes), the KEGG database(9842 genes.), the COG database (23737 genes), and the NR database (24925 genes) were obtained (**Figure 2B**). Venn diagrams present the number of genes/transcripts present in each set of samples and the overlap of genes/transcripts between groups. Transcript analysis showed that 14,418 genes in the control group and 15,149 in the treatment group were expressed. There were 13,240 co-expressed genes among them (**Figure 2C**). The differences in gene expression after 12 h of mycosubtilin homologue treatment in *Arabidopsis* seedlings were analyzed. The results showed that 6708 differentially expressed genes were detected after 12 h of mycosubtilin homologue treatment in *Arabidopsis* seedlings, of which 4336 genes were up-regulated, and 2372 genes were down-regulated (**Figure 2D**). Real-time quantitative PCR was used to validate the transcriptome data to evaluate the important transcription factors in the major signaling pathways. The results showed that the qRT-PCR expression profiles matched to the transcriptome data, indicating that the transcriptome data were reliable (**Supplementary Figure 2**).

### Transcriptome differential gene annotation and enrichment analysis

**Figure 3:**
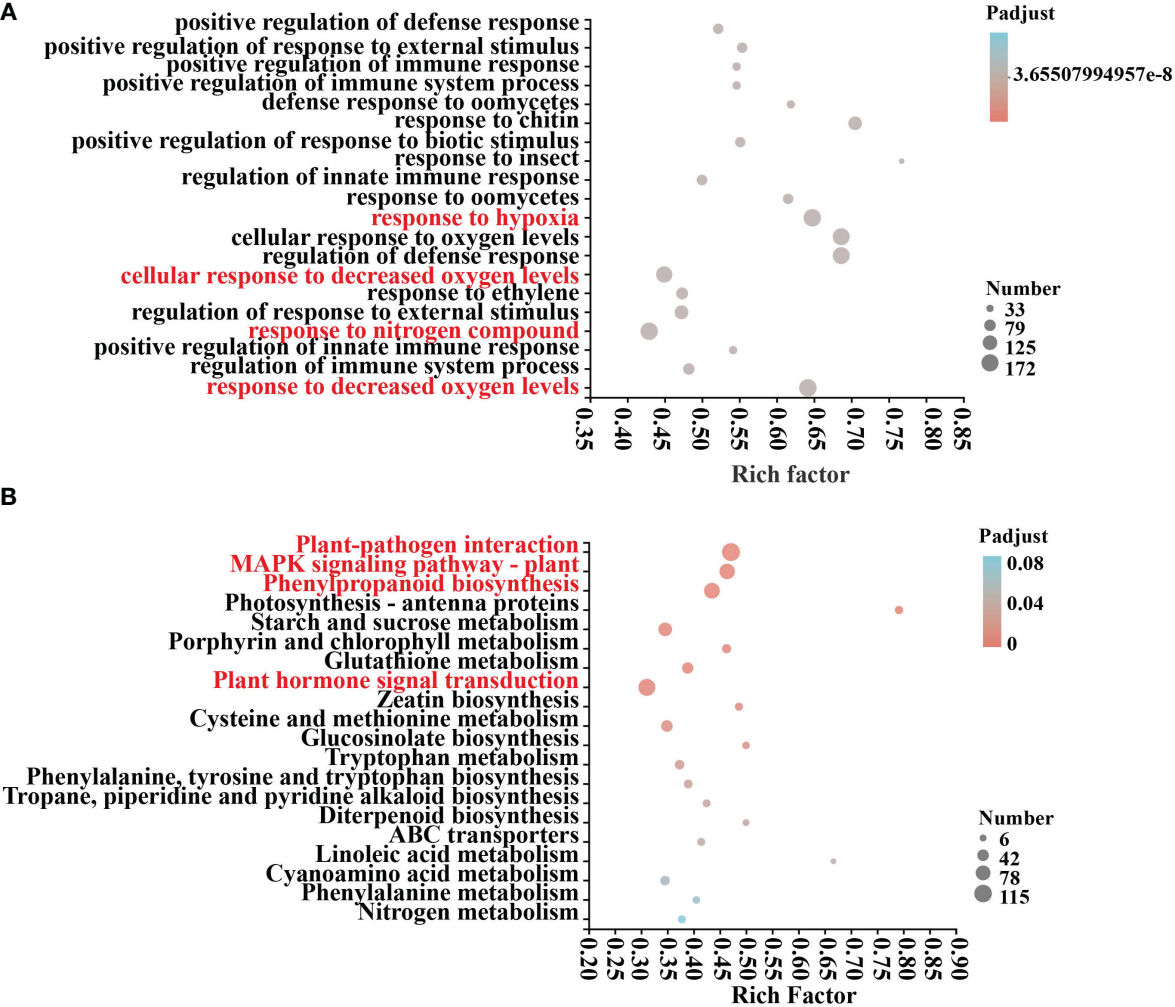
GO and KEGG differential gene enrichment analysis. **(A)** GO enrichment analysis of DEGs. **(B)** KEGG enrichment analysis of DEGs.

An examination of COG annotations of differential genes after mycosubtilin homologue treatment revealed 3659 unknown activities, 499 of which were engaged in transcription, 402 in signal transduction processes, and 386 in post-translational. The COG annotation found a total of 3659 unidentified functions, including 499 in transcription, 402 in signal transduction mechanisms, 386 in post-translational modification, protein turnover, chaperones, and 271 in carbohydrate transport and metabolism, Amino acid transport and metabolism, defense mechanisms, and other related functions (**Supplementary Table 4**). Differential genes GO annotations were classified based on the biological processes involved, the components that make up the cell, the molecular tasks they execute, and so on. After 12 hours of mycosubtilin homologue administration, GO annotations were examined in control (A) and treated (B) *Arabidopsis* seedlings. The results revealed that the main categories were cellular, metabolic, response to stimulus, biological regulation, cell parts, organelle, and membrane. The key categories were the constituent cellular components and the molecular functions achieved. The primary categories were the cell component, organelle, and membrane part, nucleotide and protein binding, catalytic activity, transcription regulator, According to the findings, the differential genes were primarily focused on several GO functions, including response to decreased oxygen levels, cellular response to decreased oxygen levels, defense response to oomycetes, positive regulation of response to external stimulus, positive regulation of immune system process, and positive regulation of innate immune response (**Figure 3A** **) (** **Supplementary Table 7**). KEGG annotation analysis of 6708 differential genes in control (A) and treated (B) *Arabidopsis* seedlings after 12 h of mycosubtilin homologue treatment. The findings revealed that the differential genes were associated with major metabolic pathways such as carbohydrate metabolism, amino acid metabolism, biosynthesis of other secondary metabolites, lipid metabolism, cofactor, and vitamin metabolism, glycan biosynthesis and metabolism, folding, sorting, and degradation, membrane transport, catabolism, and environmental adaptation (**Supplementary Table 6**). KEGG enrichment analysis of differential genes after mycosubtilin homologue treatment. The results showed that the differential genes mainly concentrated on GO functions such as Plant-pathogen interaction, MAPK signaling pathway – plant, Phenylpropanoid biosynthesis, and Plant hormone signal transduction (**Figure 3B** **) (** **Supplementary Table 8**).

### Enrichment analysis of genes and pathways related to systemic resistance and growth and development in *Arabidopsis*

**Figure 4:**
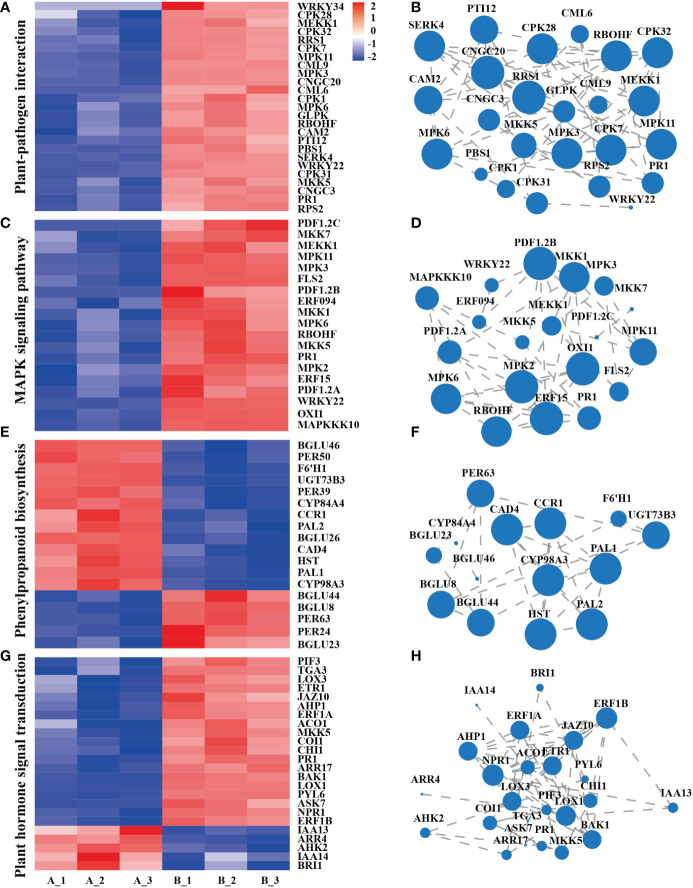
Integration analysis of genes of the major signaling pathways of the transcriptome KEGG Pathway. **(A, B)** Gene enrichment and expression correlation analysis of genes involved in the plant-pathogen interaction pathway. **(C, D)** Correlation analysis of the enrichment and expression of genes involved in the MAPK signaling pathway. **(E, F)** Gene enrichment and expression correlation analysis of the biosynthetic pathway gene of phenylpropanoid biosynthesis. **(G, H)** Gene enrichment and expression correlation analysis of plant hormone signal transduction pathways. Each node represents a gene, and the line between nodes represents the correlation of expression between genes. The larger the node, the greater the number of expression correlations between the gene and other genes. Heatmap shows FPKM values in each treatment, normalized using z-score.

Plant-pathogen interaction, the main response includes the perception of pathogens by the cell surface pattern recognition receptor (PRR). Clustering and expression correlation analysis of mycosubtilin homologue treatments showed that 115 genes were involved in the plant-pathogen interaction pathway, which was related to Ca^2+^, MAPK, reactive oxygen species, and hypersensitive responses. Among them, *PR1* is the main gene in plant disease resistance. Its expression occurred after 12 h of mycosubtilin homologue treatment in *Arabidopsis* seedlings, which is consistent with the previous results (**Figures 4A, B**). The MAPK signaling pathway, which is found in all eukaryotes, transports extracellular signals to the nucleus or cytoplasm for appropriate cellular responses. The MAPK signaling pathway was triggered after 12 hours of treatment of *Arabidopsis* seedlings with mycosubtilin homologue. A significant number of genes were activated by cascade to cause the expression of downstream growth and defense-related genes. Enrichment and expression correlation analysis of MAPK signaling pathway-related genes revealed that under mycosubtilin homologue treatment, more than 80 genes were involved in this pathway, including *PR1, RBOHF, ERF*, and *ERF*, and *PDF1.2*, which are mainly involved in plant resistance-related functions (**Figures 4C, D**). Many structural and signaling molecular mechanisms involved in phenylpropanoid biosynthesis, a class of secondary plant metabolites derived from phenylalanine, are crucialfor plant growth and development as well as plant responses to biotic and abiotic stressors. Transcriptome analysis showed that mycosubtilin homologue treatment of *Arabidopsis* seedlings for 12 h up-regulated the expression of Peroxidase, Beta-glucosidase, Cytochrome P450, Phenylalanine ammonia-lyase, Cinnamyl alcohol dehydrogenase and UDP-glycosyltransferase, which are key genes related to plant defense response and growth and development, were up-regulated this pathway (**Figures 4E, F**). Treatment with mycosubtilin homologue increased the expression of PRs family genes in the SA signaling pathway. The JA signaling pathway enhanced the expression of JAZ, JAR, LOX, AOS, and AOC family genes, while the ET signaling pathways ACS, ACO, EBF, and ERF-related genes were up-regulated. With the majority of genes in the IAA and BR signaling pathways being down-regulated in expression, other hormones were also involved in this transcriptional rearrangement (**Figures 4G, H**).

### GC-MS analysis of mycosubtilin homologue treated *Arabidopsis*

**Figure 5:**
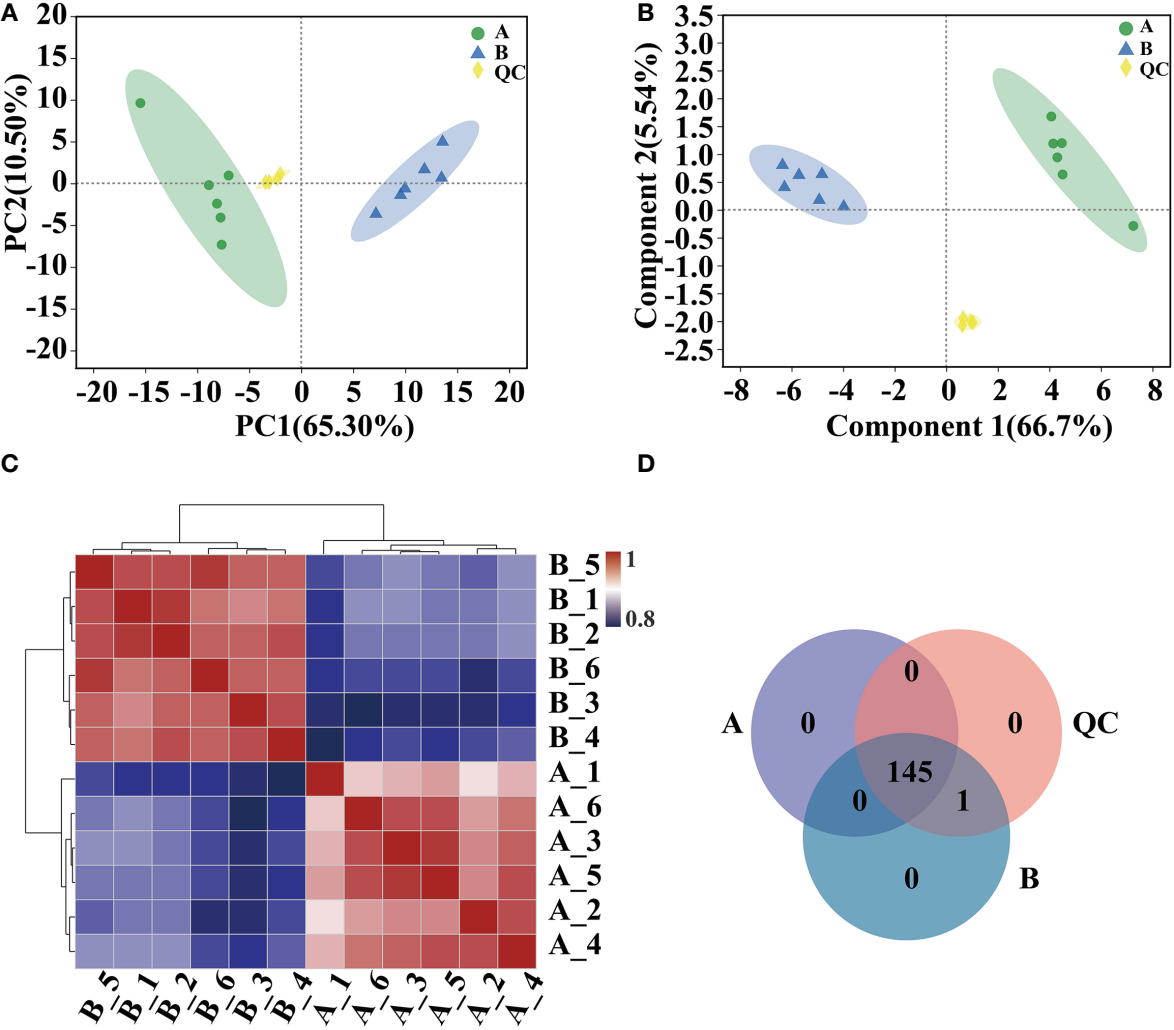
Comparative analysis of metabolic group samples. **(A, B)** Inter-sample metabolomic PCA and PLS-DA plots. **(C)** Heat map of differences between metabolic group samples. **(D)** Veen map of sample metabolites.

Analyses of PCA, PLS-DA model validation, and quantitative correlation data between samples revealed a high degree of similarity in the degree of variation in metabolite composition and abundance among various groups. Nonetheless, the degree of heterogeneity in metabolite composition and abundance between groups differed to some degree (**Figures 5A–C**). Veen analysis between groups showed that GC-MS detected a total of 145 metabolites in *Arabidopsis* 10-day-old seedlings; 146 metabolites were detected after mycosubtilin homologue treatment, 145 of which were the same as in the untreated group, and the same 146 metabolites were detected in the quality control group (**Figure 5D**).

KEGG annotation analysis was performed on 145 differential metabolites in control (A) and treated (B) *Arabidopsis* seedlings after 12 h of mycosubtilin homologue treatment. The results showed that the 145 metabolites after 12 h of mycosubtilin homologue treatment in *Arabidopsis* seedlings were localized to eight major KEGG metabolic pathways, which were identified by comparing the KEGG Compound database to the primary metabolite classes of Carbohydrates, Hormones, and transmitters, Lipids, Nucleic acids, and Nucleic acids. Lipids, Nucleic acids, Organic acids, Peptides, SteroidsVitamins, Cofactors acids, Bases Nucleic acids, Carboxylic acids, Amines, Amino acids, 29-Carbon atoms, and Vitamins, with the largest number of peptide amino acid-related compounds and the lowest number of vitamins and hormones. The primary metabolic pathways of the identified compounds were counted (top 20). The greater the number of metabolites involved, the more active the metabolic pathway. The results demonstrated that metabolites are involved in ABC transporters, Aminoacyl-tRNA biosynthesis, Alanine, aspartate and glutamate metabolism, Arginine and proline metabolism, Glyoxylate and dicarboxylate metabolism, Phenylalanine metabolism, Glycine, serine and threonine metabolism, Cyanoamino acid metabolism and beta-Alanine metabolism (**Supplementary Table 9**).

### Enrichment analysis of differential metabolites and differential metabolic pathways

**Figure 6:**
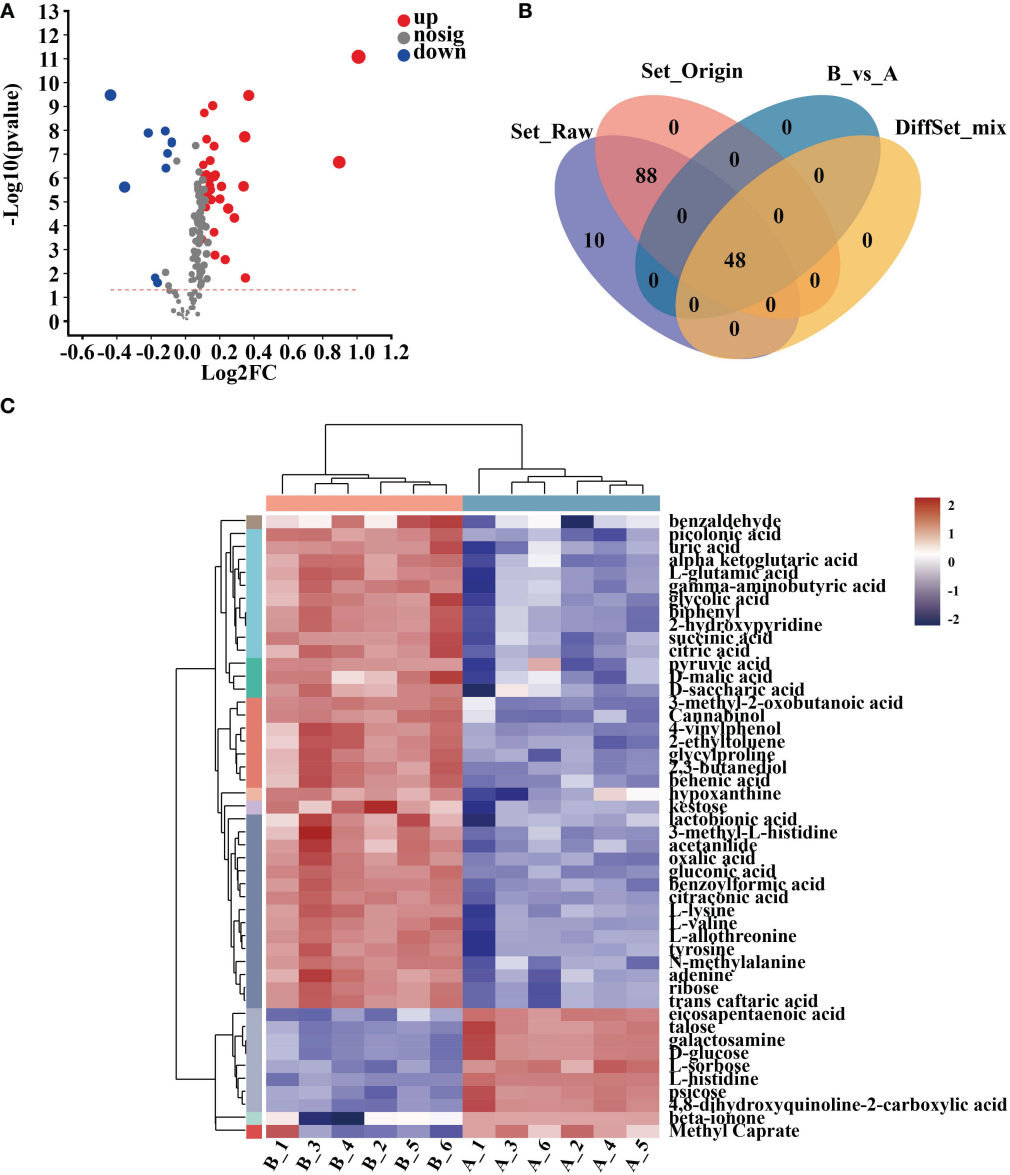
Cluster analysis of differential metabolites. **(A)** Differential metabolite volcano map. **(B)** Veen map of differential metabolites. **(C)** Heat map of differential metabolite clustering analysis.

In 10-day-old *Arabidopsis* seedlings, 48 metabolites were discovered after 12 hours of treatment with mycosubtilin homologue and two unnamed metabolites. Of these 48 compounds, nine were down-regulated, and 38 were up-regulated (**Figures 6A, B** **) (** **Supplementary Table 10**). Cluster analysis of the differential metabolites showed that benzaldehyde, picolinic acid, uric acid, α-ketoglutaric acid, L-glutamic acid, gamma-aminobutyric acid, glycolic acid, biphenyl, 2-hydroxypyridine, succinic acid, citric acid, pyruvic acid, D-malic acid, D-saccharic acid, 3-methyl-2-oxobutanoic acid, Cannabinol, 4-vinyl phenol, 2-ethyl toluene, glycylproline, 2,3-butanediol, behenic acid, hypoxanthine, kestose, lactobionic acid, 3-methyl-L-histidine, acetanilide, oxalic acid, gluconic acid, benzoylformic acid, citraconic acid, L-lysine, L-valine, L-allothreonine, tyrosine, N-methyl alanine, adenine, ribose, and trans caftaric acid were up-regulated in *Arabidopsis* seedlings after mycosubtilin homologue treatment. Eicosapentaenoic acid, talose, galactosamine, D-glucose, L-sorbose, L-histidine, psicose, 4,8-dihydroxyquinoline-2-carboxylic acid, beta-ionone, and methyl caprate were down-regulated (**Figure 6C**).

**Figure 7:**
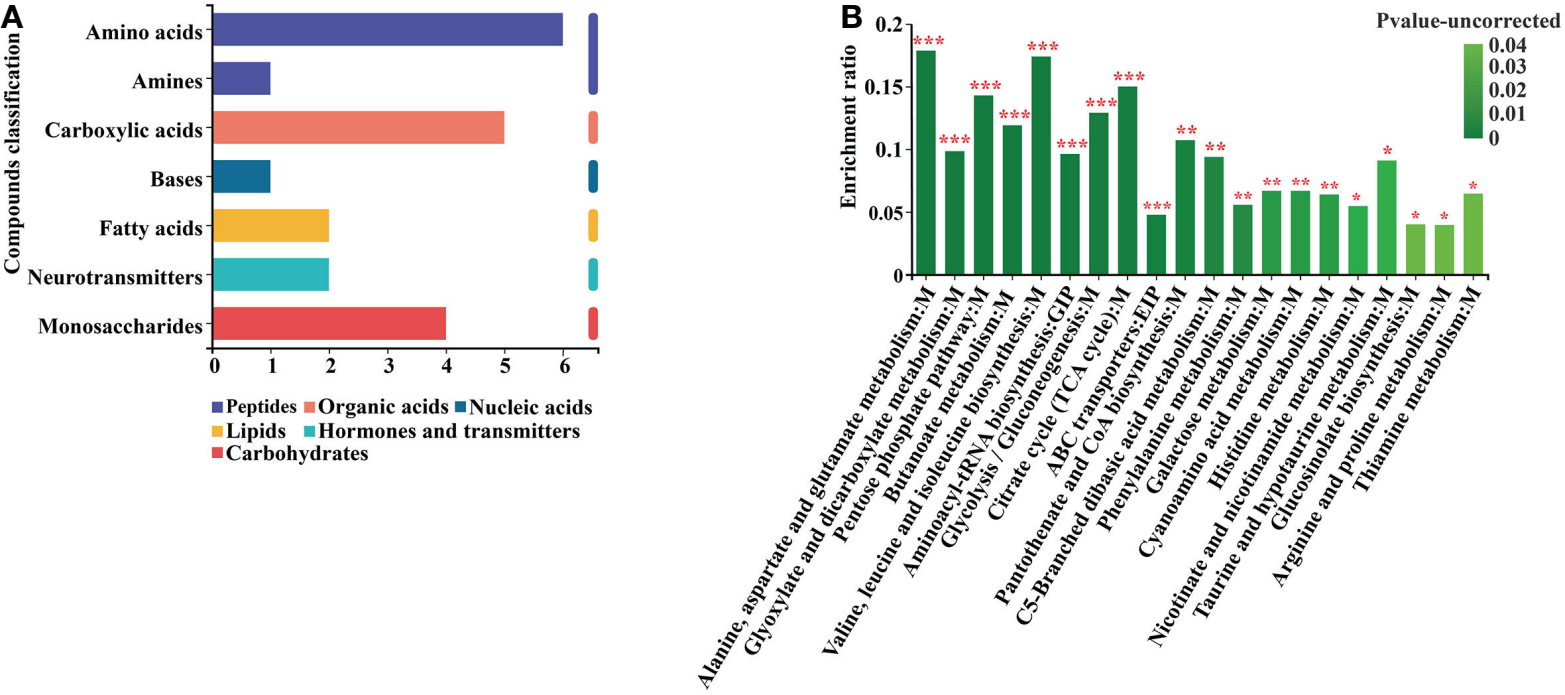
Differential metabolite KEGG analysis. **(A)** Annotated enrichment of differential metabolite KEGG compounds. **(B)** Analysis of differential metabolite importance. *, ** and *** indicates statistical significance at p < 0.05, p < 0.01 and p < 0.001, respectively (Student’s t-test).

According to the KEGG classification of the differential metabolites based on their biological effects, they were mostly Peptides, Organic acids, Nucleic acids, Lipids, Hormones, Transmitters, and Carbohydrates (**Figure 7A**). The results of the enrichment analysis of the metabolic pathways in which the differential metabolites were involved showed that the resulting differential metabolites were mainly enriched in Alanine, aspartate and glutamate metabolism, Pentose phosphate pathway, Glyoxylate and dicarboxylate metabolism, Butanoate metabolism, Valine, leucine and isoleucine biosynthesis, Aminoacyl-tRNA biosynthesis, Glycolysis and Gluconeogenesis, Citrate cycle, ABC transporters, Phenylalanine metabolism, and Pyruvate metabolism metabolic pathways (**Figure 7B** **) (** **Supplementary Table 11**).

**Figure 8:**
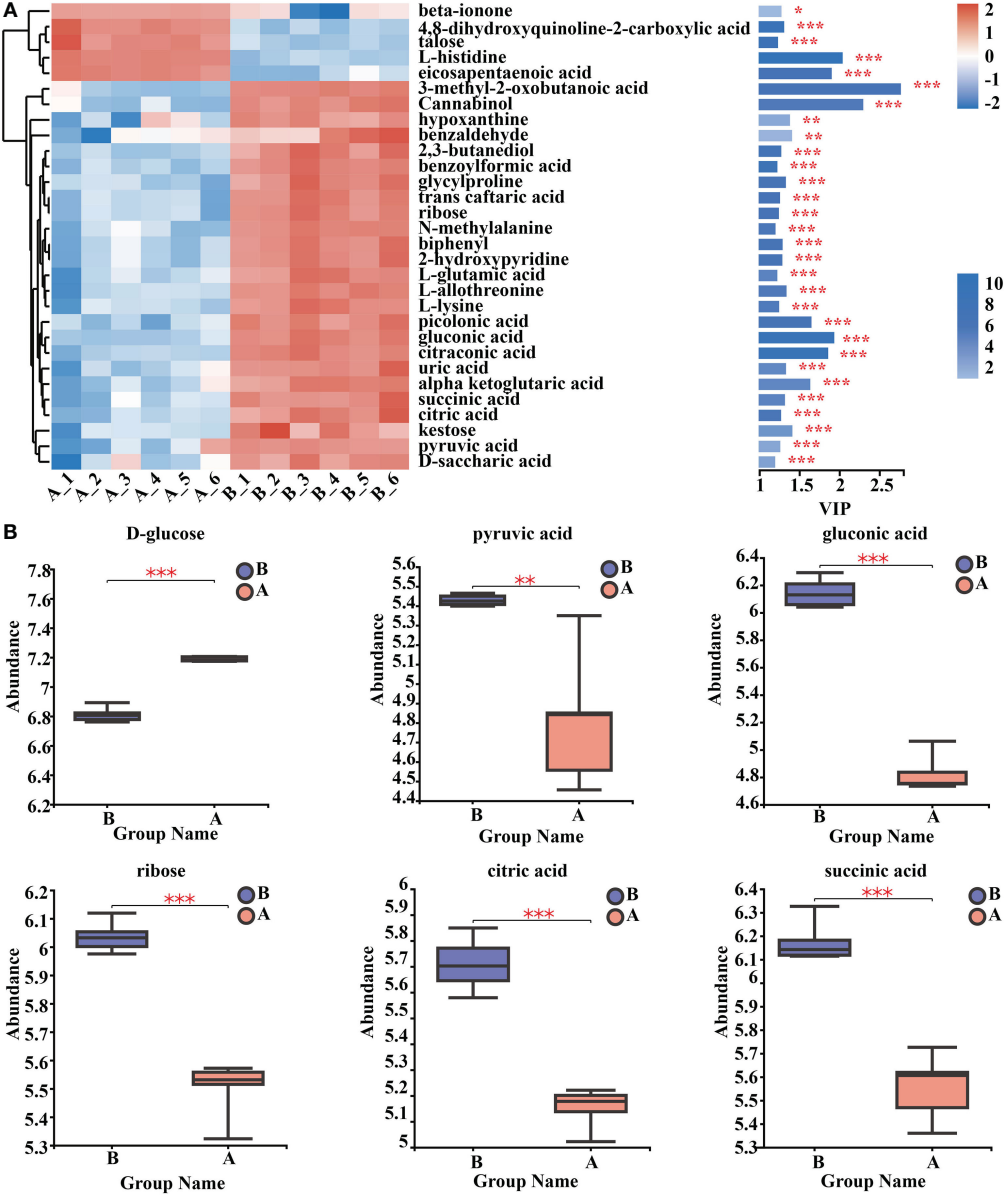
Analysis of key differential metabolites. **(A)** Metabolomics key differential metabolites enrichment. The smaller the p-value, the larger the -log_10_ (p-value), and the darker the colour. **(B)** Relative expression abundance of key differential metabolites. The Y coordinate is the mass spectral intensity value (after data pre-processing). *, ** and *** indicates statistical significance at p< 0.05, p < 0.01 and p < 0.001, respectively (Student’s t-test).

By using clustering heat maps and VIP bar charts to assess the p-values of metabolites in VIP and unidimensional statistics of multivariate statistical analysis, it was possible to depict changes in the significance and expression trends in differential metabolites. Mycosubtilin homologue treatment significantly increased the expression of metabolites in Amino acid metabolism, Carbohydrate metabolism, Lipid metabolism, and Energy metabolism in *Arabidopsis* seedlings (**Figure 8A** **) (** **Supplementary Table 12**). For instance, the amount of gluconic and pyruvic acids produced during glycolysis, ribose produced during the pentose phosphate pathway, and citric and D-malic acids produced during the tricarboxylic acid cycle were all reduced (**Figure 8B** **) (** **Supplementary Table 9**).

## Discussion

Previous studies have shown the efficacy and potential mechanism of *B. subtilis* BS-Z15 to inhibit various plant pathogens such as *V. dahliae*. Previous studies have been conducted on cotton to control *V. dahliaee* (Lin et al., 2022). This study investigated the BS-Z15 secondary metabolite mycosubtilin homologue a novel plant immune activator that regulates plant defense responses. We established, a bioinformatic network of mycosubtilin homologue-triggered plant immune activation by integrating RNA-seq and GC-MS analyses. The results showed that a mycosubtilin homologue regulates plant defense systems through various metabolic and signaling pathways. We found that a mycosubtilin homologue controls plant defense mechanisms *via* various metabolic and signaling pathways.

It has been demonstrated that *B. subtilis* produces lipopeptides, including surfactants, iturin, and fengycins, that provide it with the potential to manage plant infections through biocontrol (Ongena and Jacques, 2008). In the present study, the biocontrol of plant pathogens by BS-Z15 was demonstrated to be associated with its secondary metabolite, mycosubtilin homologue. The structural diversity of LPs produced by *Bacillus* suggests that these metabolites may have different modes of action to enhance plant immunity (Ongena et al., 2007). The potential of LPs to activate the ISR in plants has been extensively researched. However, little is known about the molecular mechanisms behind the local defense response after LP activation. The iturin family member, a mycosubtilin homologue, is shown to have excitonic capabilities for the first time in the current study. Mycosubtilin homologue activates a multilayered ISR defense response in *Arabidopsis* with defense gene expression, ROS burst and accumulation, callose deposition, and MAPK (MPK3/MPK6) cascade phosphorylation. It enables *Arabidopsis* to resist pathogenic bacteria *V. dahliaee* and *PstDC3000* mycosubtilin homologue induced defense responses resembling the typical MAMPs bacterial flagellin flg22 were all inhibitory to plant root length (Chinchilla et al., 2006), which may be related to plant homeostasis system resistance and growth and development.

Plant cells may recognize mycosubtilin homologue-triggered ISR and SAR by receptor identification or by perturbing the plasma membrane bilayer by an unidentified method. This recognition will direct plant cells to initiate early defense response processes. When the SA signaling route is active, the expression of the disease resistance gene *PR1* is increased, and when the JA signaling pathway is involved, the expression of the defense gene *PDF1.2* is triggered. On iturin can act as an activator of plant defense responses, several studies have shown that iturinA-induced plant defenses should be somewhat targeted. This may be because LPs only act as initiators in specific plant species (Kawagoe et al., 2015). Mycosubtilin homologue was used as an initiator activity in the current study to activate systemic resistance in *Arabidopsis*. It is unknown if particular receptor proteins that bind mycosubtilin homologue to exist in plant cells, even though mycosubtilin homologue can cause systemic resistance in plants. Iturin A targets and inhibits the pro-angiogenic/invasive factors VEGF and MMP-2/9 in animal cells to bind MD-2/TLR4 (Dey et al., 2017).

However, iturin binds strongly to phytosterols. Ion permeability is greatly enhanced in cells in which iturin is present by forming ion-conducting pores using the β-hydroxy fatty acid chains of iturin (Dana and Peypoux, 1994). Plants do not appear to have any LPs-binding receptors. According to studies, surfactants bind to lipid fractions and alter their stability, which causes plants to respond defensively (Henry et al., 2011). The interaction of mycosubtilin with the phospholipid membrane has also been demonstrated. However, it is yet unknown if this is connected to the plant immune response (Nasir et al., 2010). Fatty acid chains have also been reported to be key to inducing systemic resistance in plants (Kutschera et al., 2019), so the ability of mycosubtilin homologue to act as an excitant in a given plant species may depend on the β-hydroxy fatty acid chain of the substance, or it may be due to the structure of the plant cells themselves.

In this study, a transcriptional regulatory network was built for the synergistic regulation of systemic resistance and growth and development by mycosubtilin homologues to investigate the mechanism of systemic resistance induced by LPs in plants. *Arabidopsis* seedlings were treated with mycosubtilin homologues for 12 hours, and the key genes that changed included WRKY, MYB, NAC, bHLH, etc. After treatment, these genes’ dynamic expression affected how the plant responded to biotic and abiotic stimuli and systemic resistance. Notably, mycosubtilin homologue affects metabolic processes like *Arabidopsis* plant-pathogen interactions, plant MAPK signaling pathways, phenylpropanoid biosynthesis, and phytohormone signaling. The MAPK cascade is a highly conserved signaling module downstream of the receptor/sensor that translates extracellular stimuli into intracellular responses in eukaryotes. The plant MAPK cascade plays a key role in signaling plant defense against pathogenic and in signaling plant defense against pathogen attacks (Meng and Zhang, 2013). This pathway involved more than 80 genes, including *PR1* and *PDF1.2*, after mycosubtilin homologue treatment, indicated that the MAPK signaling pathway plays an important role in regulating systemic resistance and growth development by mycosubtilin homologues in *Arabidopsis*. Most plant secondary metabolites are derived from the phenylpropanoid synthesis pathway, which plays an important role in plant growth, development, and response to adversity stress (Fraser and Chapple, 2011). 12 h treatment of A large number of genes in this signaling pathway are induced to be expressed, suggesting that the phenylpropanoid synthesis pathway is important for plant responses to external substances. Several studies have shown that SA, JA, and ET are inextricably linked to microbially induced plant ISR (Pieterse et al., 2012; Yang et al., 2015). 12 h treatment of *Arabidopsis* seedlings with mycosubtilin homologue resulted in up-regulated expression of many genes in the SA, JA, and ET signaling pathways. These findings support the hypothesis that iturin and fengycins induce ISR synthesis in rice at the hormone transcript level (Paiboon et al., 2019). Moreover, after mycosubtilin homologue treatment, genes involved in the IAA and BR signaling pathways were markedly down-regulated, and mycosubtilin homologue inhibited the growth of *Arabidopsis* primary roots, suggesting that mycosubtilin homologue may be controlled by phytohormone levels in promoting plant systemic resistance, growth, and development.

Mycosubtilin homologue can control plant systemic resistance, growth, and development in *Arabidopsis* through various signaling pathways, according to a GC-MS study. Then, we built a bioinformatics network using a mycosubtilin homologue to control metabolic growth and development and systemic resistance in *Arabidopsis*. Mycosubtilin homologue-treated *Arabidopsis* seedlings significantly increased the content of metabolites related to some energy metabolic pathways. Glycolysis, the pentose phosphate pathway, and the citric acid cycle are plants’ main energy sources for metabolic changes. Their accessibility has a significant bearing on plant development and might be connected to the induction of defensive mechanisms in plants. The manufacture of many precursors involved in other secondary metabolic pathways may rise as this class of metabolites increases. During plant defense, the secretion of malic acid recruits probiotic bacteria, and after mycosubtilin homologue treatment, *Arabidopsis* thaliana malic acid and citric acid are over-secreted. After being treated with a mycosubtilin homologue, the plant may have developed a strong dynamic defense (Rudrappa et al., 2008; Lakshmanan et al., 2012). Studies have also observed that alcohols and polyols undergo dynamic changes under the influence of mycosubtilin homologues in *Arabidopsis*. The accumulation of alcohol and polyol metabolites have been reported to be associated with plant tolerance to biotic and abiotic stresses (Stoop et al., 1996; Saddhe et al., 2021). As a result, mycosubtilin homologue therapy could improve plants’ ability to withstand abiotic stressors. L-phenylalanine, benzoic acid, and caffeic acid, among other precursors of the phenylpropanoid synthesis pathway, were considerably elevated in mycosubtilin homologue-treated *Arabidopsis* seedlings, according to GC/MS analyses. The treatment of *Arabidopsis* seedlings with a mycosubtilin homologue greatly impacted the dynamic balance of amino acid metabolism. Mycosubtilin homologue affected several amino acid metabolic pathways in *Arabidopsis* seedlings, inducing changes in several of these amino acids. Many studies have shown that these metabolic pathways are essential for plant growth and stress response (Shulaev et al., 2008). In a recent study, fengycin was shown to stimulate seeds alone to regulate lipid metabolism and glutathione accumulation, providing protection to plants and controlling the development of plant radicles (Clavero et al., 2022). Thus, it is clear that lipopeptides are key factors in the induction of systemic resistance in plants by *Bacillus*. The construction of a complex regulatory network will facilitate the better exploitation of such substances and provide new insights into plant protection.

## Conclusion

In conclusion, concerning plant interactions, the regulation of systemic resistance and the growth and development of *Arabidopsis* by the secondary metabolite BS-Z15 mycosubtilin homologue was examined. In order to better understand the mechanisms underlying the interactions between *B. subtilis* and plants, as well as the systemic dynamics of transcription and metabolism of typical MAMPs inducing plant immune responses, a comprehensive bioinformatics network architecture of the action of the lipopeptide mycosubtilin homologue on plants were constructed by transcriptome and metabolome.

## Data availability statement

The datasets presented in this study can be found in online repositories. The names of the repository/repositories and accession number(s) can be found in the article/**Supplementary Material**.

## Author contributions

All authors contributed to the study’s conception and design. HXZ and HPZ conceived and designed the experiments. QY, HZ, and JYo performed the experiments and wrote the articles. JYa and QZ helped to perform the experiments and collected the data. JJZ and RA participated in the statistical analysis. BZJ helped with the chart processing. HXZ and SH contributed to the manuscript discussion and revision. All authors read and approved the final manuscript. 
