# Supplementary figures and images for: Transcriptome and metabolome analyses reveal that Bacillus subtilis BS-Z15 lipopeptides mycosubtilin homologue mediates plant defense responses
Source: Front Plant Sci. 2023 Feb 6;13:1088220. doi: 10.3389/fpls.2022.1088220 (PMC9940755; doi:10.3389/fpls.2022.1088220)

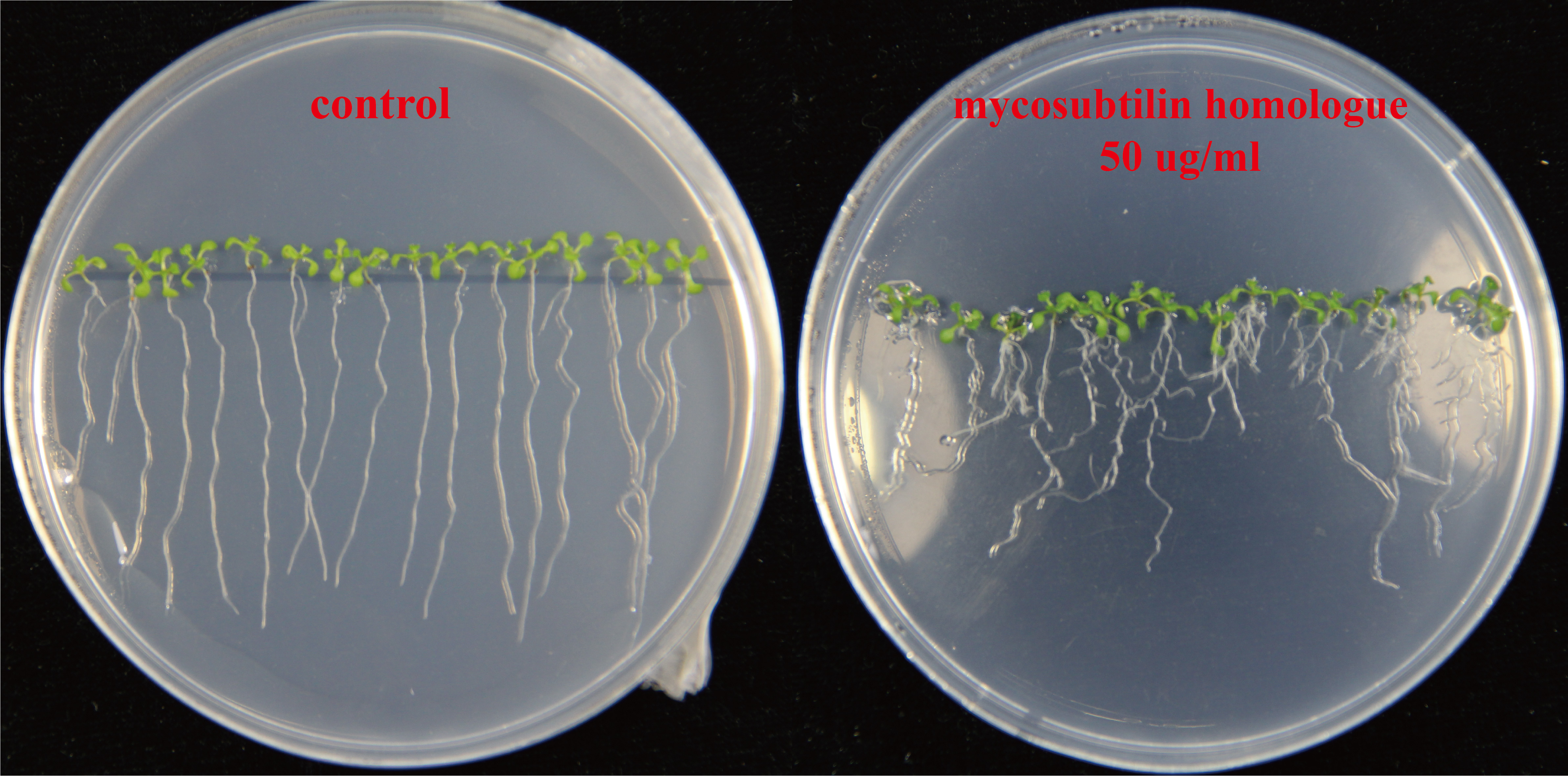

Supplement: Supplementary file 1 [file Image_1.jpeg]

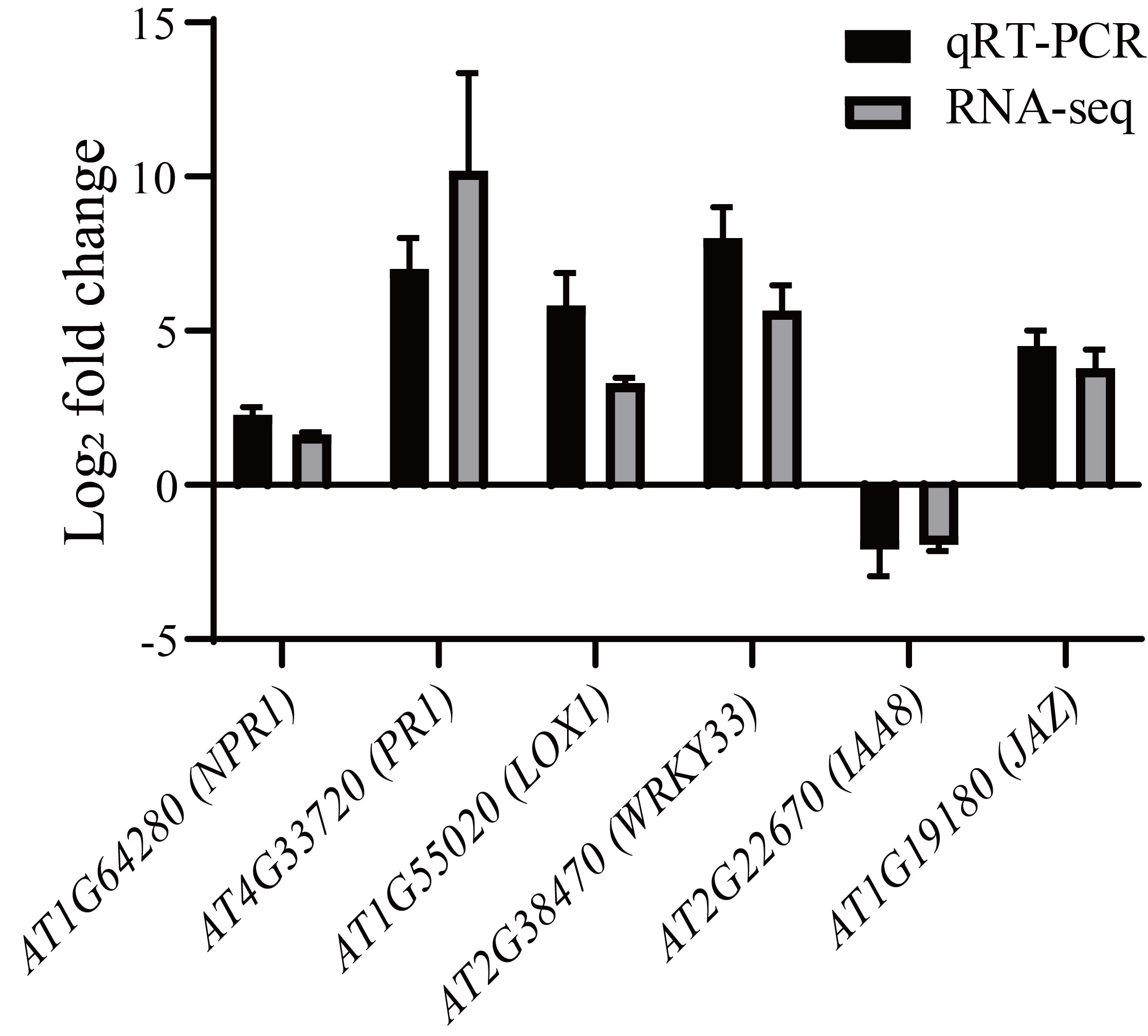

Supplement: Supplementary file 2 [file Image_2.jpeg]
